# Alterations of choroidal circulation and vascular morphology in a patient with chronic myeloid leukemia before and after chemotherapy

**DOI:** 10.1186/s12886-022-02380-4

**Published:** 2022-04-07

**Authors:** Mizuho Mitamura, Satoru Kase, Kiriko Hirooka, Hiroaki Endo, Yuki Ito, Yuko Cho, Susumu Ishida

**Affiliations:** 1 Department of Ophthalmology, Faculty of Medicine and Graduate School of Medicine, Hokkaido University, N-15, W-7, Kita-ku, Sapporo, 060-8638 Japan; 2 Department of Ophthalmology, Teine Keijinkai Hospital, Sapporo, Japan; 3 Department of Pediatrics, Hokkaido University, Sapporo, Japan

**Keywords:** Chronic myeloid leukemia, Leukemic retinopathy, Laser speckle flowgraphy, Optical coherence tomography, Binarization method

## Abstract

**Background:**

Chronic myeloid leukemia (CML) is known to cause leukemic retinopathy due to leukemia cell invasion into the choroid; however, details of the circulatory dynamics and morphological changes in the choroid are unknown. The aim of this study was to present a case of leukemic retinopathy and examine choroidal circulatory and structural analyses using laser speckle flowgraphy (LSFG) and optical coherence tomography with a binarization method, respectively.

**Case presentation:**

A 15-year-old male diagnosed with CML complained of blurred vision in his right eye. He was ophthalmologically diagnosed with leukemic retinopathy due to retinal hemorrhage in both eyes. Tyrosine kinase inhibitors achieved complete cytogenetic remission and resolution of retinal hemorrhages at 6 months after treatment. After the treatment, the best-corrected visual acuity had recovered from 0.1 to 1.2 oculus dexter (OD) and remained at 1.5 oculus sinister (OS). The rate of change in macular blood flow assessed by the mean blur rate on LSFG was 18.3% increase OD and 25.2% decrease OS 19 months after treatment. The central choroidal thickness showed 0.4 and 3.1% reductions OD and OS, respectively. The binarization technique demonstrated that the rate of luminal areas in choroidal areas exhibited 3.2% increase OD but 4.8% decrease OS.

**Conclusion:**

Choroidal blood flow improved OD after treatment for CML, while it deteriorated OS, together with choroidal thinning due to reduction of luminal areas. The degrees of leukemia cell invasion into the choroidal tissue and tissue destruction might be different between the eyes in this case.

## Background

Chronic myeloid leukemia (CML) is known to cause leukemic retinopathy due to leukemia cell invasion into the retino-choroidal tissues [1]. The choroid is the most common site of leukemia cell invasion in the eye, and histopathology of postmortem eyes revealed various forms of choroidal invasion in 65% of leukemia patients [1]. Moreover, histopathological examination revealed markedly thickened choroids compared with normal choroids at the posterior pole, where leukemia cells invaded choroidal vessels and stroma [1]. Choroidal vascular invasion may be divided into intravascular and/or extravascular invasion, the former of which indicates that choroidal vascular lumens are filled with tumor cells. The latter predominantly shows leukemia cell invasion in the choroidal stroma [2]. The pathology-proven choroidal thickening in acute CML may be consistent with central choroidal thickness (CCT) thickening based on optical coherence tomography (OCT) reported previously [2]. Previous clinical studies demonstrated that choroidal thicknesses revealed 8.8–37.0% reduction after chemotherapy for CML compared with before treatment [3, 4].

Recently, binarization of enhanced depth imaging (EDI)-OCT images has facilitated quantitative assessments of luminal and stromal areas in the choroid over time noninvasively [5, 6]. Indeed, histological intravascular and extravascular areas could correspond to the binarized luminal and stromal areas using EDI-OCT, respectively [7]. In hematological malignancies, Egawa et al. reported reductions of stromal areas, but not luminal areas, after treatments in five cases of primary intraocular lymphoma (PIOL), suggesting that EDI-OCT depicted predominant stromal invasion rather than vascular involvements of PIOL cells [8]. However, little is known about the alterations of choroidal vascular structures in leukemic retinopathy.

Laser speckle flowgraphy (LSFG) is a blood flow imaging device based on laser scattering, which non-invasively allows for two-dimensional visualization of fundus circulation. We have employed LSFG, looking at fundus circulations in various intraocular tumor(−like) lesions such as optic disc melanocytoma [9], choroidal macrovessel [10], sclerochoroidal calcification [11], juxtapapillary retinal capillary hemangioblastoma [12], and choroidal lymphoma [13]. In addition, Takita et al. used LSFG and EDI-OCT imaging to identify choroidal hypoperfusion and choroidal thickening in the acute stage of CML, both of which improved with remission by chemotherapy [4]. Based on the previous studies, we hypothesized that choroidal circulation may be synchronously involved with choroidal morphological alterations in patients with CML following treatments. However, the correlation between circulatory dynamics and vascular structural changes in the choroid has not been fully elucidated.

We herein present a case of leukemic retinopathy and report choroidal circulatory and structural analyses using LSFG and OCT with the binarization method, respectively.

## Case presentation

**Fig. 1:**
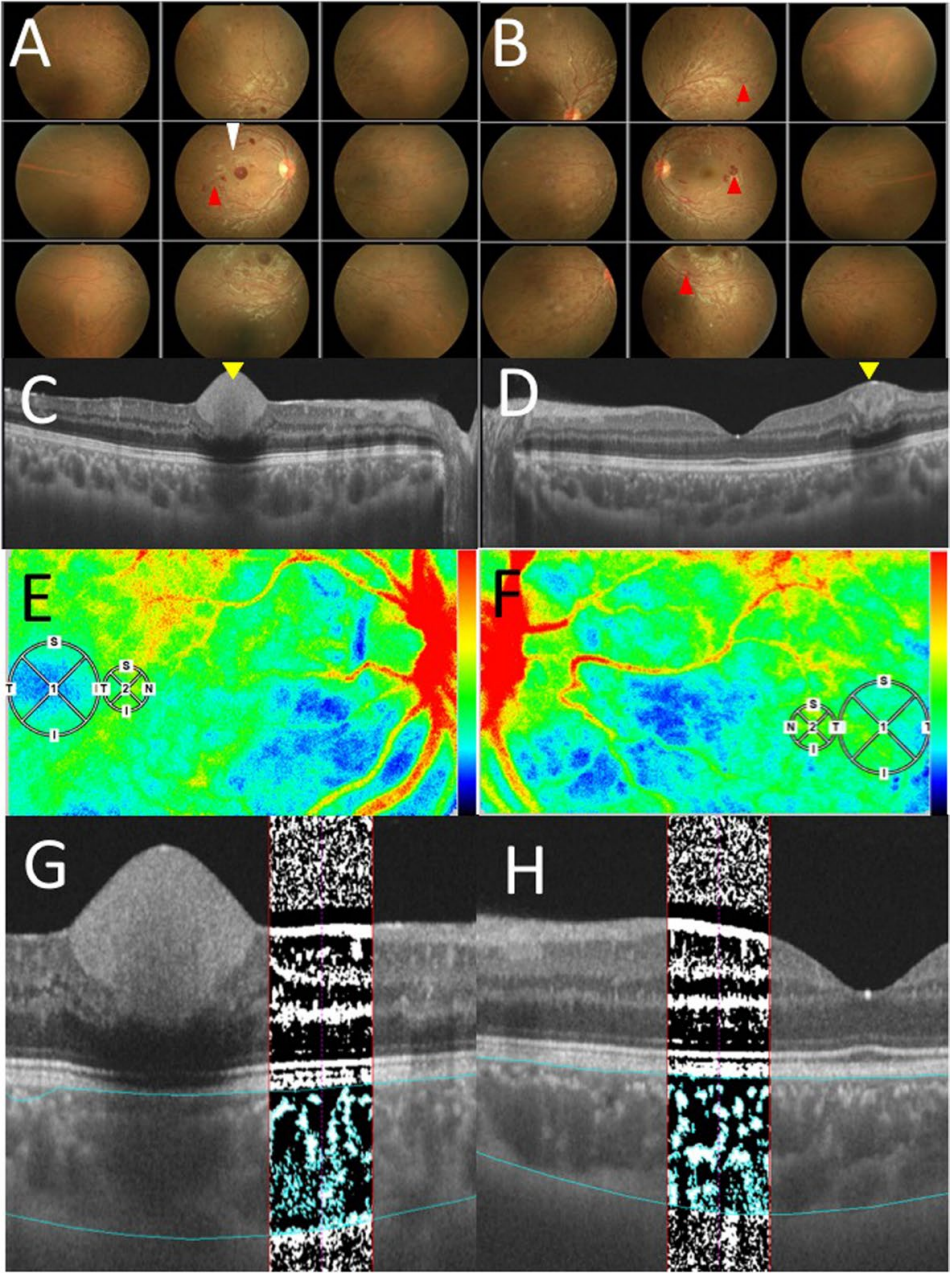
Initial findings on color fundus photography (CFP), swept-source optical coherence tomography (SS-OCT), laser speckle flowgraphy (LSFG), and OCT images with the binarization method in the present case of leukemic retinopathy. **A-B** CFP showed multiple mottled hemorrhages, Roth spots (red arrowheads), and dilated tortuosity of retinal veins OU as well as sub-internal limiting membrane hemorrhage at the macula OD (white arrowhead). **C-D** SS-OCT on horizontal scans through the fovea revealed hyperintense reflection consistent with hemorrhages OU (yellow arrowhead). **E-F** LSFG showed a mild warm-color blood flow signal corresponding to the small circles OU. (Large circles corresponding to the fovea). **G. H.** In OCT images with the binarization method, bright and dark pixels correspond to the stromal and luminal regions of the choroid, respectively

**Fig. 2:**
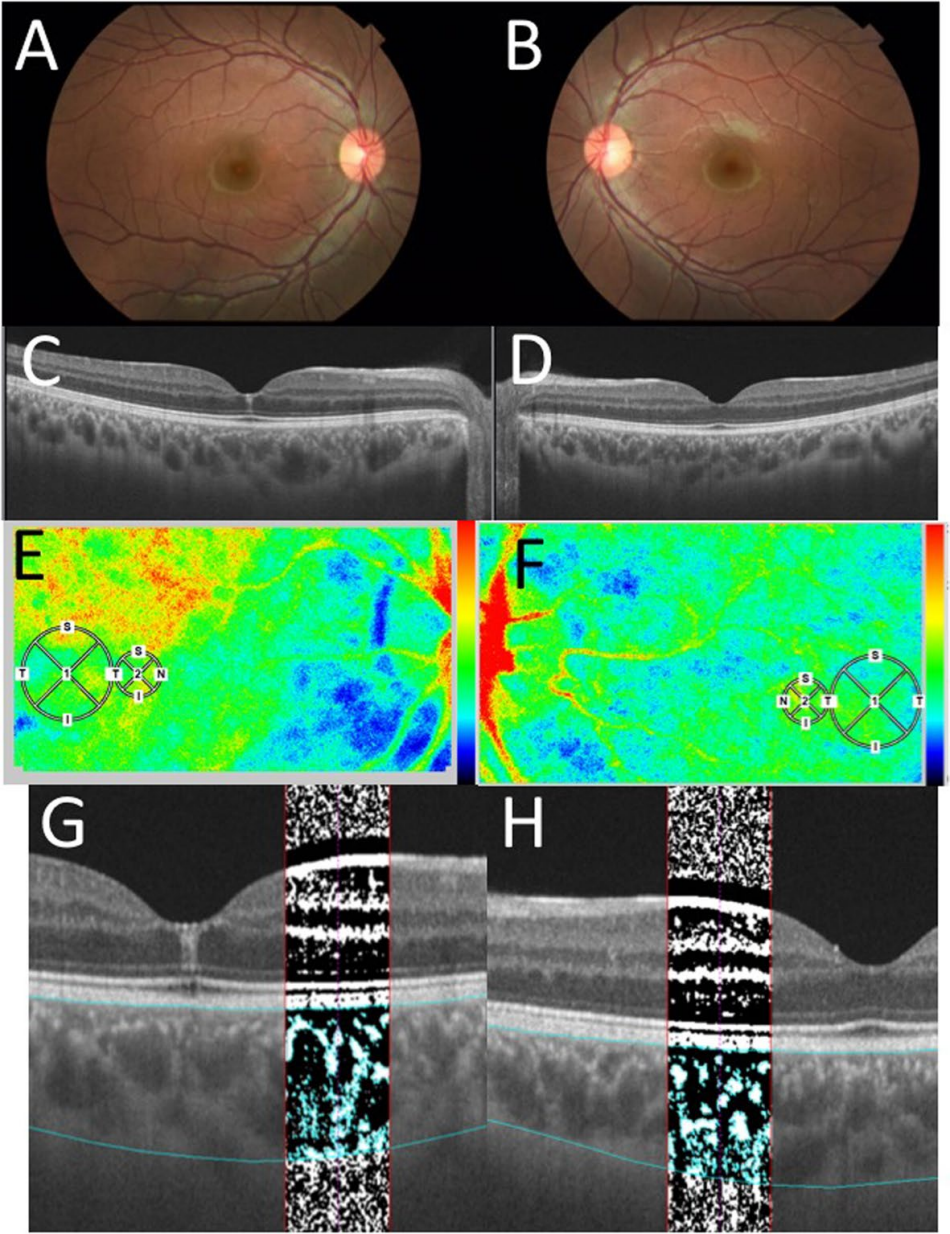
Ophthalmic findings after 6 months of treatment. **A-B** CFP in the right eye showed that the mottled hemorrhages had disappeared and the tortuosity of the veins had improved. OU. **C** SS-OCT on horizontal scans through the fovea revealed a slight residual hyperintense lesion in the central fovea OD. **D** SS-OCT on horizontal scans through the fovea revealed normal OS. **E-F** LSFG showed a mild warm-color blood flow signal corresponding to the small circles OU. **G-H** OCT images with the binarization method showed changes in the choroidal structure OU

A 15-year-old Japanese male complained of blurred vision in his right eye and was referred to our clinic because of bilateral retinal hemorrhage. The left eye had no symptoms of vision loss. There were no special notes on the medical or family history. He was frequently aware of muscle strain during exercise. His best-corrected visual acuity (BCVA) was 0.1 oculus dexter (OD) and 1.5 oculus sinister (OS), with normal intraocular pressure oculi uterque (OU). Slit-lamp microscopy did not detect any findings OU. Color fundus photography showed multiple mottled hemorrhages, Roth spots, and dilated tortuosity of retinal veins OU and sub-internal limiting membrane (ILM) hemorrhage at the macula OD (Fig. 1A and B). Swept-source (SS)-OCT on horizontal scans through the fovea revealed hyperintense reflections consistent with the lesions OU (Fig. 1C and D; yellow arrowheads). A blood test showed a high white blood cell count of 350,000 (/μL), and systemic examinations including bone marrow biopsy by a pediatrician was performed. Bone marrow biopsy showed abnormal hyperplasia (nucleated cell count: 48.7 × 10^4^/μL) with a high percentage of myeloid cells (myeloid erythroid ratio: 162.33) in various stages of differentiation without dysplasia. Bcr-abl was positive in 99% of cells with fluorescence in situ hybridization, and major bcr-abl was detected with reverse transcription-polymerase chain reaction. These results led to a diagnosis of *BCR-ABL*-positive CML. Based on the clinicopathological findings, this patient was ophthalmologically diagnosed with leukemic retinopathy. Two days after the first visit to our department, Imatinib mesylate was started immediately after diagnosis in the pediatric department. Due to severe bilateral knee pain causing insomnia, Imatinib mesylate was switched to Dasatinib hydrate on day 113. However, Dasatinib hydrate had to be discontinued due to remarkable elevation of serum creatine kinase, and had to be switched further to Nilotinib hydrochloride hydrate on day 147. These treatments resulted in cytogenetic complete remission after 6 months of the treatments. His BCVA was 1.2 OU, and retinal hemorrhages and hyperintense reflections on OCT disappeared after 6 months of treatment (Fig. 2A-D). His BCVA was unchanged, and the fundus and OCT showed no marked abnormalities 19 months later. The institutional review board of Hokkaido University waived ethical assessment of this clinical study because it was a single case report with a non-invasive study. This study adhered to the tenets of the Declaration of Helsinki. Briefly, the basic fundamental principle was respect for the individual (Article 8), his right to self-determination, and the right to make informed decisions (Articles 21 and 22) regarding participation in research, both initially and during the course of the research. In addition, due to the young age of the individual, his mother accompanied him to obtain informed consent (Article 20) [14].

This study further analyzed choroidal circulatory and structural alterations using multi-modal imaging. First, this study evaluated the alterations of choroidal blood flow after chemotherapy using LSFG. Relative blood flow was determined as the mean blur rate (MBR), following a quantitative measure of blood flow velocity using LSFG software (LSFG-NAVI, version 3.1.39.2, Softcare Ltd., Fukuoka, Japan), in accordance with previous reports [4, 11]. The pupils of the subject were dilated with 0.4% tropicamide (Mydrin-M; Santen Pharmaceutical Co., Ltd., Osaka) before the tests. Ophthalmic examinations were conducted after the pupils were completely devoid of reflex in both eyes. The central fovea was manually marked in the MBR images, and the vessels were automatically segmented by the system’s software (LSFG Analyzer, version 3.0.47.0) using an automatically defined threshold. To avoid the area of sub-ILM hemorrhage at the macula OD, a circle of 750 μm in diameter nasal to the sub-ILM hemorrhage OD and to the fovea OS was defined as the region of interest on LSFG (Fig. 1E, F, 2E, and F: small circles). Four to five consecutive measurements were made for each circle, and the mean values were used for analysis. All examinations were performed by a single experienced operator. Ocular perfusion pressure (OPP) was calculated using the patient’s blood pressure and IOP, as previously described [15].

**Fig. 3:**
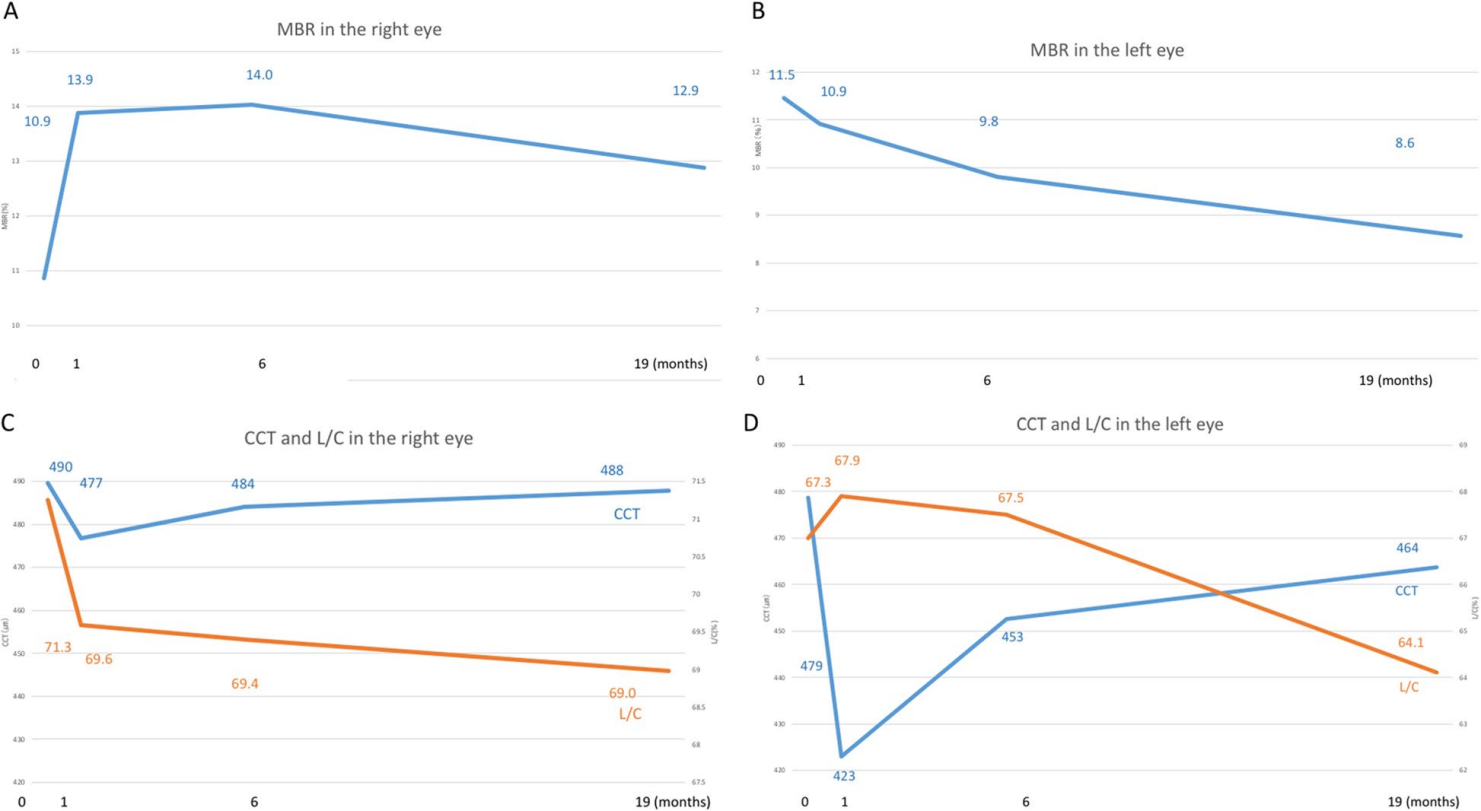
Mean blur rate (MBR) by laser speckle flowgraphy (**A**,** B**) and the central choroidal thickness (CCT) and luminal area/choroidal area (L/C) ratio (**C**,** D**). **A.** MBR increased temporarily after initiation of chemotherapy, and remained almost the same after remission OD. **B** MBR gradually decreased from the time of initial diagnosis to after the start of chemotherapy, and was progressive OS. **C** CCT decreased temporarily after the start of chemotherapy, but increased after remission OD. The L/C ratio decreased from the time of initial diagnosis after the start of chemotherapy, and did not recover after remission OD. **D** CCT decreased temporarily after the start of chemotherapy, but increased after remission OS. The L/C ratio gradually decreased from the time of initial diagnosis after the start of chemotherapy, and did not recover after remission OS

The MBR values OD are shown in Fig. 3A as follows: 10.9, 13.9, 14.0, and 12.9 at the initial visit, and 1, 6, and 19 months after the initial visit, respectively. MBR of the right eye increased immediately after the start of treatment and remained unchanged thereafter. The rate of change assessed by MBR was 18.3% increase OD 19 months after treatment. The MBR values OS are shown in Fig. 3B as follows: 11.5, 10.9, 9.8, and 8.6 at the initial visit, and 1, 6, and 19 months after the initial visit, respectively. The rate of change assessed by MBR was 25.2% decrease OS 19 months after treatment. OPP was 49.3, 36.0, 34.8, and 42.8 mmHg OD and 47.8, 35.9, 31.3, and 39.8 mmHg OS at the initial visit, and 1, 6, and 19 months after the initial visit, respectively, revealing no significant changes in either eye.

Next, the CCT in SS-OCT was measured manually from the lower edge of the retinal pigment epithelium layer to the scleral border by two experienced examiners. The choroidal structure of SS-OCT images (DRI OCT Triton; Topcon Inc., Tokyo, Japan) was analyzed using EyeGround, a semi-automated analysis software developed by Sonoda et al. [6], by selecting the 750 μm area of the OCT images as the region of interest corresponding to the LSFG analysis range. In EyeGround, the luminance of each pixel in the OCT images was binarized using the Niblack method. The bright and dark pixels in the generated images corresponded to the choroidal stromal and luminal regions, respectively (Fig. 1G and H at initial visit; Fig. 2G and H after 6 months in our case). The area of both regions was measured using the binarization method, which was quantitatively assessed. Choroidal area (CA), luminal area (LA), stromal area (SA), and the ratios of the luminal area/choroidal area (L/C) and stromal area/choroidal area (S/C) were measured by two experienced examiners and the average values are shown.

The CCT values OD were 490, 477, 484, and 488 μm at the initial visit, and 1, 6, and 19 months after the initial visit, respectively (Fig. 3C). The CCT values OS were 479, 423, 453, and 464 μm at the initial visit, and 1, 6, and 19 months after the initial visit, respectively (Fig. 3D). CCT of both eyes decreased immediately after the start of treatment, and then gradually increased thereafter. The rates of change in choroidal structures by the binarization method in the designated 750 μm circle 19 months after treatment were as follows: L/C increased by 3.2% OD (Fig. 3C) and decreased by 4.8% OS (Fig. 3D); LA increased by 3.8% OD and decreased by 8.3% OS; CA increased by 9.8% OD and decreased by 2.3% OS; SA increased by 18.6% OD and increased by 6.4% OS.

## Discussion and conclusion

The present study demonstrated differences of choroidal circulatory dynamics and vascular morphology after chemotherapy in both eyes with leukemic retinopathy by LSFG and the binarization method. This case revealed a reduction in CCT of both eyes after treatment, which was consistent with the previous report by Takita et al. [4], whereas the decrease in MBR in the left eye of this case was different. For the right eye, CCT showed a 1.2% reduction 1 months later compared with the initial visit, which gradually increased at 6 months. The L/C ratio, an index of vascular morphology, decreased over 6 months. The decreases in CCT and the L/C ratio indicated that the thickened choroid with vessel dilatations before treatment was reduced together with resolution of tumor invasion after starting chemotherapy. There might be two mechanisms underlying the reduction of choroidal vascular structures: the disappearance of leukemia cells from within the vessels, and the improvement of congestion caused by hyperviscosity associated with a very high leukocyte number. Furthermore, the improvement in MBR after chemotherapy indicated that the function of the choroidal vascular system was restored. Since MBR of the right eye increased after the start of systemic treatments with CCT reduction, the choroidal abnormality is consistent with “a pseudo-inflammatory pattern” [4]. The gradual recoveries of CCT and MBR together with increased SA until 19 months suggested structural remodeling in the choroid following systemic treatments.

In contrast, for the left eye, the 5.4% reduction rate in CCT after chemotherapy, which was greater than that in the right eye, suggested that tumor invasion may be much broader within the choroidal tissues. In contrast to the right eye, the decreases in MBR as well as the L/C ratio were prominent following treatment in the left eye. Fukutsu et al. reported a case of diffuse large B-cell lymphoma with choroidal invasion showing low MBR throughout the clinical course in parallel with choroidal thinning after chemotherapy, suggesting that choroidal invasion by neoplastic cells caused persistent and irreversible damage to the vasculature [13]. We recently reported that MBR and the L/C ratio were progressively reduced following carbon iron beam radiation therapy for choroidal melanoma, suggesting that choroidal vascular damage by irradiation of the globe was irreversible [16]. Similarly, in the left eye of our case, MBR gradually decreased over time and did not recover, suggesting persistent and irreversible choroidal blood circulatory disturbance due to severe tissue destruction by leukemia cell invasion.

This multi-modal imaging study also compares with previous pathological findings in autopsy eyes. Reduction in the L/C ratio following chemotherapy in the right eye may be consistent with vessel dilatations at pretreatment due to hyperviscosity and/or intra/extravascular invasion of leukemia cells observed in the pathology of autopsy eyes [2]. MBR improved together with L/C ratio reduction after treatment, which correlates with recovery of hyperviscosity with preservation of choroidal circulation rather than vascular tissue destruction by leukemic cell invasion. Instead, extravascular invasion could take place in the choroid rather than intravascular invasion, where leukemia cells might not have disrupted endothelial functions. On the other hand, the concurrent decreases in MBR and the L/C ratio in the left eye reflect structural and functional disturbance of choroidal vessels, which may exclusively result from intravascular invasion by leukemia cells. The marked invasion into the choroidal vascular lumen shown by the pathological findings [2] might have led to intravascular endothelial damage and subsequently irreversible circulatory disturbance, even after treatment in this case. The changes in choroidal circulation in both eyes shown by LSFG may thus reflect pathological findings in autopsy eyes.

In conclusion, choroidal blood flow improved OD before and after treatment for CML, while it deteriorated OS, together with choroidal thinning due to reduction of vascular lumens. The degrees of leukemia cell invasion into the choroidal vascular tissue might be different between the eyes in this case.

## Data Availability

The datasets used and analyzed in the current study are available from the corresponding author on reasonable request.

## Abbreviations

CML: Chronic myeloid leukemia
CCT: Central choroidal thickness
PIOL: Primary intraocular lymphoma
LSFG: Laser speckle flowgraphy
ILM: Internal limiting membrane
BCVA: Best-corrected visual acuity
OD: Oculus dexter
OS: Oculus sinister
OU: Oculi uterque
SS: Swept-source
MBR: Mean blur rate
OPP: Ocular perfusion pressure
CA: Choroidal area
LA: Luminal area
SA: Stromal area
L/C: Luminal area/choroidal area
S/C: Stromal area/choroidal area
